# Adding open spectral data to MassBank and PubChem using open source tools to support non-targeted exposomics of mixtures†

**DOI:** 10.1039/d3em00181d

**Published:** 2023-07-10

**Authors:** Anjana Elapavalore, Todor Kondić, Randolph R. Singh, Benjamin A. Shoemaker, Paul A. Thiessen, Jian Zhang, Evan E. Bolton, Emma L. Schymanski

**Affiliations:** a Luxembourg Centre for Systems Biomedicine (LCSB), University of Luxembourg 6 Avenue du Swing 4367 Belvaux Luxembourg anjana.elapavalore@uni.lu emma.schymanski@uni.lu; b IFREMER (Institut Français de Recherche pour l’Exploitation de la Mer), Laboratoire Biogéochimie des Contaminants Organiques Rue de l’Ile d’Yeu, BP 21105 Nantes Cedex 3 44311 France; c National Center for Biotechnology Information (NCBI), National Library of Medicine (NLM), National Institutes of Health (NIH) Bethesda MD 20894 USA

## Abstract

The term “exposome” is defined as a comprehensive study of life-course environmental exposures and the associated biological responses. Humans are exposed to many different chemicals, which can pose a major threat to the well-being of humanity. Targeted or non-targeted mass spectrometry techniques are widely used to identify and characterize various environmental stressors when linking exposures to human health. However, identification remains challenging due to the huge chemical space applicable to exposomics, combined with the lack of sufficient relevant entries in spectral libraries. Addressing these challenges requires cheminformatics tools and database resources to share curated open spectral data on chemicals to improve the identification of chemicals in exposomics studies. This article describes efforts to contribute spectra relevant for exposomics to the open mass spectral library MassBank (https://www.massbank.eu) using various open source software efforts, including the R packages RMassBank and Shinyscreen. The experimental spectra were obtained from ten mixtures containing toxicologically relevant chemicals from the US Environmental Protection Agency (EPA) Non-Targeted Analysis Collaborative Trial (ENTACT). Following processing and curation, 5582 spectra from 783 of the 1268 ENTACT compounds were added to MassBank, and through this to other open spectral libraries (*e.g.*, MoNA, GNPS) for community benefit. Additionally, an automated deposition and annotation workflow was developed with PubChem to enable the display of all MassBank mass spectra in PubChem, which is rerun with each MassBank release. The new spectral records have already been used in several studies to increase the confidence in identification in non-target small molecule identification workflows applied to environmental and exposomics research.

## Abstract 2

Adding mass spectra from complex mixtures of chemicals relevant for exposomics to open spectral libraries such as MassBank and chemical databases like PubChem empowers identification efforts for non-target high resolution mass spectrometry workflows.

## Graphical abstract

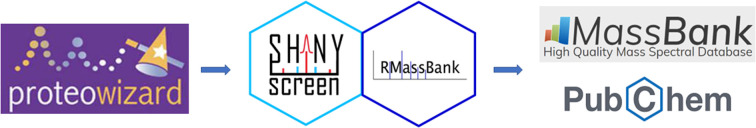

**Environmental significance statement**

Humans are exposed to countless chemicals each day, some of which may pose a major threat to human health and/or the environment. Identifying these chemicals can be especially challenging since they are present in the environment as complex mixtures, not individual chemicals. Thus, it is important to provide the research community with open source cheminformatics tools and databases to support identification efforts. This article presents an open source workflow for setting up an open mass spectral collection of toxicologically relevant chemical compounds available in complex chemical mixtures, combined with propagating this information further to other open resources to support international efforts to protect human health and ecosystems.

## Introduction

The environment plays an essential role in the well-being of humanity. However, humans and all living beings are subjected to different chemical exposures, which can result in direct and indirect consequences on human health and the environment. This raises concerns about the impacts of exposures over our lifetime, making it imperative to study chemical exposure to recognize the effects on humans and ecosystems.^1^ Furthermore, the volume and number of chemicals in use are increasing drastically.^2^ Not all chemicals have immediate or obvious health effects. Some emerging contaminants (ECs) may effect humans and the environment even at low doses, while many effects go unnoticed until they reach a critical level to cause a given health issue. It is possible that the effects may not be seen for many years or decades. One such example is Parkinson's disease.^3^ ECs comprise a diverse group of chemicals including pesticides, pharmaceuticals, endocrine disruptors, personal care products, surfactants, flame retardants, plasticisers, industrial agents, artificial sweeteners, and gasoline additives.^4^

Chemicals can interact in numerous possible ways, making it difficult to predict the effects of different chemical mixtures.^5^ So far, epidemiological studies have generally focused on single factors but sidelined the combined effect of different chemical exposures.^6^ Due to poor characterization of exposures and growing awareness of the need to study several exposures simultaneously, interest in the concept of the exposome is increasing.^7^ The term exposome was first proposed by Christopher Wild in 2005, when he defined it as the “life-course environmental exposures (including lifestyle factors), from the prenatal period onwards”.^8,9^ Studies on the exposome can encompass small molecules (*e.g.*, lipids, drugs, xenobiotic compounds, metabolites) as well as non-chemical stressors such as radiation, industrial processes, consumer goods, pathogens and diet. In exposome analysis, mass spectrometry (MS) is a widely used analytical technique, mainly due to its suitability to perform a sensitive qualitative and quantitative analysis of complex samples. MS, when coupled with gas or liquid chromatography for separation before mass spectrometric detection,^10^ covers a wide range of applications by separating out sample complexity and providing additional structural information about the compounds as they elute from a chromatographic column. Methods based on gas chromatography coupled with MS (*i.e.*, GC-MS) analysis have been developed to analyze different classes of chemical substances, including highly hydrophobic and volatile substances. However, more polar pesticides, water-soluble endocrine disruptors and industrial pollutants are more amenable to liquid chromatography (LC)-MS methods.^11^ LC-MS, especially high resolution MS (HR-MS), is widely used for screening emerging pollutants and the detection of different chemicals can rely on many different tools.^12^ The high throughput omics technologies (metabolomics/exposomics) have helped in integrating a wide range of exposures.^7^ Advancements in analytical instruments, high throughput statistical analysis, HR-MS, data processing algorithms, chemical compound databases and mass spectral libraries contribute to exposome research.^13^ Ideally, this would lead to the identification of chemical stressors and robust biomarkers that can be used to deduce the adverse effects of exposures.^14^

Mass spectrometry analysis can be broadly categorized into two different approaches: (i) targeted analysis and (ii) non-targeted analysis (NTA). In targeted analysis, the compounds (so-called “targets”) are known in advance and can be identified in the sample through optimized laboratory methods developed with the use of reference standards. This method requires prior knowledge of the compounds and suitable methods to be developed for the targeted approach.^15^ However, ECs are often overlooked in samples when only targeted analysis is performed, since it is impossible to perform target analysis on the multitude of ECs potentially present in the environment. This can be addressed using a non-targeted approach to identify “unknowns” in the sample.^16^ Compound-specific data is collected *via* instrumental analysis (‘detection’) and the detected mass spectrometric feature is then linked with the tentative chemical identity based on the evidence, in a process called ‘annotation.’ The process of verifying that the annotated compound is indeed the proposed chemical is called ‘identification’.^17^ Despite all these steps in identifying the compounds, the peaks cannot always be interpreted with the same confidence level.^18^ To report identification confidence more accurately, a five-level scheme ranging from level 1(confirmed) to level 5 (exact mass only) is often used to report the identification confidence level.^19^ Confident identification of unknown chemical substances in MS studies requires consistent workflows and corresponding computational tools and data. To increase the identification confidence, it is vital to use experimental evidence combined with chemical metadata, library searching and chemistry databases in the workflow. A mass spectral library will facilitate level 2a identification confidence by providing a sufficient match of the probable structure with the library spectrum. A level 1 confidence can be achieved when the retention time (RT) of the data acquired in the same analytical setup in-house matches with the library spectrum.^19^ Supported by such information, NTA methods can use advanced analytical instruments, spectral libraries and computational tools to help identify “known unknowns” or previously understudied compounds. Thus, NTA methods can be used to screen a broad spectrum of chemicals occurring in the environmental samples, which is essential to explore the exposome. This method does not need any prior knowledge about the chemicals to be screened and allows rapid chemical characterization of chemicals.^15^

A typical NTA workflow includes several steps: sample collection, sample preparation, analysis and data processing. However, each of these steps becomes a tedious process – especially the data treatment step. The data processing steps mainly involve peak picking, peak alignment, peak integration and finally identifying the structure of the compound behind the peaks.^18^ Thus, creating an in-house spectral library will improve future NTA workflows internally, potentially to level 1. Additionally, uploading the spectra online will facilitate level 2a identifications at other institutes. Mass spectral libraries are capable of providing rapid tentative identifications with a relatively high level of confidence in this regard.^20^ MassBank was established in 2006 and published in 2010 as one of the first open mass spectral libraries, hosted in Japan.^21^ MassBank Europe was established as a mirror server in 2012.^22^ Over the years the code and data migrated to GitHub,^23^ making MassBank a truly open access, open data, open source resource. As such, the spectral records included in MassBank are available for use by researchers in various analytical and computational workflows. MassBank is an important resource of spectra for metabolomics, environmental and exposomics studies. MassBank records are text files containing compound metadata and mass spectral information in the rich MassBank record format.

Since mass spectral libraries are inherently incomplete and the fragmentation spectrum of a given chemical typically only conveys a fraction of the chemical information, it is challenging to ascertain the identity of a chemical in NTA using fragmentation information alone. Additionally, compound (*i.e.*, chemical) databases can be used to more accurately identify and annotate chemicals in a sample. It is possible to search compound databases for candidates by using the exact mass or calculated molecular formula, combined with *in silico* fragmentation techniques to sort the candidates.^20^ The National Institutes of Health (NIH) maintains PubChem, an open chemistry database containing more than 115 million compounds (https://pubchem.ncbi.nlm.nih.gov/).^24^ PubChem is one of the most comprehensive open online chemical databases. Data in PubChem is available for download in multiple formats (including CSV, XML, JSON, and SDF) and is complemented by a range of web services and APIs. PubChem offers many features for researchers and scientists in a variety of disciplines. As the community moves beyond pure chemical identification towards more detailed interpretation of HR-MS datasets, improving the content in publicly available databases is imperative.

In August 2015, EPA's Non-Targeted Analysis Collaborative Trial (ENTACT) was initiated to evaluate the efficiency of NTA methods for identifying unknown chemicals in samples. The purpose of this trial was to generate, interpret and exchange different NTA results and set up a database of spectral records that can be used for future NTA evaluation in identifying unknown compounds. ENTACT offered a way to obtain large numbers of chemicals of various classes for method development, as well as a means to test various workflows. This study aimed to generate MassBank records for the ENTACT mixtures, to expand the internal LCSB spectral library as well as the public MassBank.EU library, to support future non-target studies on complex mixtures of chemicals. This article describes the methods and results of the data processing, including the uploading of the spectra to MassBank, plus the subsequent integration studies on complex mixtures of chemicals within PubChem using open workflows.

## Methods

### ENTACT mixture composition

The ENTACT dataset contains 1268 substances (in total) sourced from individual ToxCast chemical substances, which were distributed into 10 mixes.^1^ The ten synthetic mixtures (mixes 499, 500, 501, 502, 503, 504, 505, 506, 507, and 508) contain 95–365 substances in each mix with varied levels of complexity, as indicated in Table 1. Mixes 1–4 and 9 contained a total of 95 unique substances, mixes 5 and 6 contained 185 substances each, of which mix 5 included the replicate set of mix 1, while mixes 7, 8 and 10 each contained 365 substances. Mix 7 comprised 270 substances plus the replicate set from mix 1. Mix 9 was especially designed to be the most challenging mix due to the presence of many isobaric substances (substances with identical mass – defined further below), while mix 10 contained substances of low purity and low concentration,^1^ posing a complementary analytical challenge to the other higher quality mixes.

**Table 1:** Summary of different ENTACT mixes, number of substances and complexity of the mixes

No.	Serial no.	Mix	No. of substances	Complexity
1	BF00173499	499	95	Low
2	BF00173500	500	95	Low
3	BF00173501	501	95	Low
4	BF00173502	502	95	Low
5	BF00173503	503	185	Medium
6	BF00173504	504	185	Medium
7	BF00173505	505	365	Medium
8	BF00173506	506	365	Medium
9	BF00173507	507	95	High
10	BF00173508	508	365	High

The ToxCast list provided with the ENTACT mixes contained the following information, which was used for curating the input compound list:

• Identifiers: DSSTox substance (DTXSID) and compound (DTXCID) identifiers from CompTox,^25^ and the Chemical Abstract Service (CAS) registry number;^26^

• Structure in the Simplified Molecular Input Line Entry System (SMILES) format;^27^

• InChIKey (the hash of the International Chemical Identifier, InChI^28^);

• Preferred name, molecular formula and exact mass;

• MS-ready forms^29^ of SMILES, InChIKey, molecular formula and exact mass.

An internal identifier (termed “Identifier”) was also assigned to map candidates to the correct MS/MS record in RMassBank. The “MS-ready” form refers to a structure that has had counter-ions and charges removed to represent a neutral form that will be analyzed in the instrument (pre-ionization).^29^

### Chemical analysis

Following sample receipt, an initial test analysis was conducted on the ENTACT mixtures on 12 August 2019 and remeasured on 3 March 2020. Reverse Phase Liquid Chromatography (RPLC) was performed using an Acquity UPLC BEH C_18_ column (dimensions: 1.7 μm, 2.1 × 150 mm) from Waters with a guard column. The flow was set at 0.20 mL min^−1^ using water with 0.1% formic acid (A) and methanol (B) as mobile phase. The gradient started at 90% of A and 10% of B at 0 min until 2 min before reaching 100% at 15 min. Mass spectrometric detection was performed with a Q Exactive Orbitrap HF (Thermo Scientific) in positive and negative ionization mode, using electrospray ionisation (ESI). The product ion (MS/MS) spectra were acquired in data-dependent mode using a mass list of all the mixtures as an inclusion list. The MS/MS spectra for the samples were fragmented at 6 different nominal collision energy (NCE) levels (15, 30, 45, 60, 75, and 90 NCE) in separate runs, generating data files for individual collision energies per mixture and mode.

### Data analysis

**Fig. 1:**
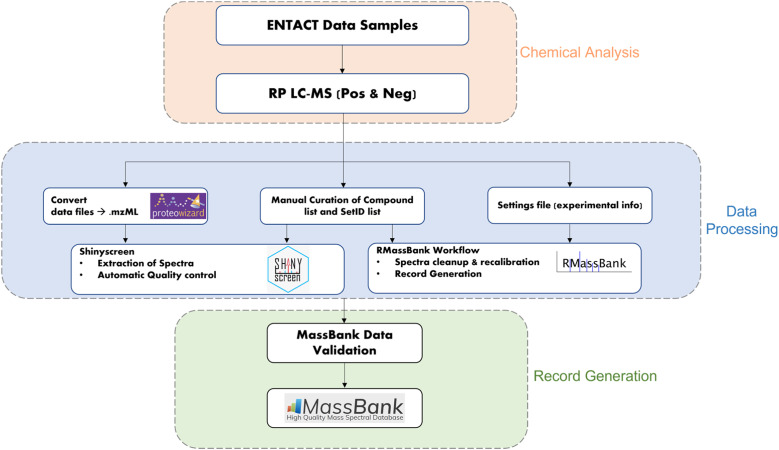
Workflow for generating MassBank records from ENTACT mixtures.

The workflow for the creation of MassBank records from the ENTACT mixtures is shown in Fig. 1. The samples and chemical analysis (shown in orange) are described above. The data extraction (blue middle section) was performed using the R packages Shinyscreen^30^ and RMassBank^31^*via* the wrapper RMB-mix,^32^ as explained in the next sections. The generated records were then validated using ReSOLUTION and the MassBank validation checks prior to upload (green section, Fig. 1). All packages and code are available on the ECI GitLab pages (see “Data availability” and “References”).

### Data extraction, prescreening and quality control with Shinyscreen

Shinyscreen is an interactive web interface developed using the shiny package in R, building on the MSnbase^33^ and mzR packages to explore, process and interpret MS data. It is specifically designed to extract the spectral data, perform an automated quality check (based on peak alignment, retention time shift, intensity and signal to noise ratio) and visualize the MS1 chromatograms plus MS/MS scans and spectra. The automated quality analysis can be updated manually after visual inspection. Shinyscreen, currently at v1.3.21, is a semi-automated computational tool to reduce the efforts of manual prescreening.^34^ This work was performed using an older version of Shinyscreen (v0.51).

The first step in the data analysis was to convert raw data files from the LC-MS analysis to the mzML file format^35^ using MSConvert from Proteowizard (v.3.0.19182-51f676fbe)^36^ in peak picking mode to convert the spectral information from profile to centroid mode. The chemical analysis resulted in mzML files per mix, mode (positive and negative) and per collision energy. These were configured as input data files in Shinyscreen, along with the ‘compound list’ for the appropriate mix (defining the chemicals to screen) and the ‘setID list’ (used to map the files, compounds and mixes). The compound list contained the unique identifiers (IDs) assigned to the ENTACT compounds, along with the name and MS-ready SMILES, saved as Comma Separated Value (CSV) file. SetID is a CSV file that includes the ID and a set (mix name), which is used to identify the compounds in each mix. The input lists are provided in the GNPS repository^37^ (see “Data availability”). The input files are configured in the configuration tab of the Shinyscreen shiny web interface. Fig. S1† shows the configuration of the ENTACT mix 499 data in negative mode: the set mix499_M (minus) represents mix 499 in negative mode and the tag represents the collision energies. The uploaded .mzML files were assigned their respective modes and collision energies in Shinyscreen and the configuration was saved.

The Shinyscreen v0.51 workflow involved the extraction of MS/MS (MS2) spectra for each compound in the data files. Following the configuration of the input files, an initial file table was generated with predetermined filtering options appropriate for the instrument (intensity threshold = 10^5^, signal to noise ratio = 3, MS2 peak = present) to automate the quality control checks. The data was prepared for spectral extraction using preset parameters for extraction and subsequent quality control. On the spectral extraction tab of the Shinyscreen web interface (Fig. S2†), the ppm was set to +/−2.5, and the retention time tolerance was set to +/−0.5 minutes before the extraction was initiated. The MS/MS scan was deemed to be RT shifted (too far from the MS1 apex) when it exceeded the RT tolerance limit and was thus excluded from further consideration. Once the threshold values were established, the data was preprocessed to perform automatic quality control analysis. Preprocessing generates a file table in CSV format, which is updated and saved at the end of the process. The compounds that failed to meet the quality criteria were excluded from further processing with the RMasSBank workflow. The automatic quality control procedure is presented in greater detail with plots demonstrating how the procedure would proceed in a “pass” and “fail” scenario elsewhere.^38^ Prescreening the mass spectrometry data is crucial to remove spectra resulting from compounds that do not satisfy the quality criteria, especially in the case of complex mixtures as measured here.

### Record generation with RMassBank and RMB-mix

Spectra that satisfied the Shinyscreen quality criteria were further processed with the RMassBank workflow for record generation, *via* the RMB-mix package.^32^ RMB-mix is a Shinyscreen-assisted RMassBank workflow, which uses the prescreening summary output to exclude compounds of bad quality prior to generating MS/MS spectral records that users can then upload to MassBank. This package adapted the RMassBank workflow for a high-throughput record generation workflow of mixtures, since the original RMassBank was designed to work on single standards. In the RMassBank package, functions include extracting tandem MS spectra, assigning formulas to fragments, calibrating tandem MS spectra with fragments, cleaning spectral data, retrieving compound information from online databases, and exporting compound information to MassBank.^31^ In addition to the mzML files and compound list, this workflow requires an additional input file known as a settings file. The settings file contains information like ionization mode, authors, instrumentation type, solvent gradient, chromatographic specification, *etc.* This is saved as ‘settings.ini’ and defines the information included in the generated MassBank records. An example settings file is provided in the GNPS repository associated with this article^37^ (see also “Data availability”).

The RMB-mix method package runs on a slightly modified version of the RMassBank (ver. 2.99.2) code to handle complex mixtures like ENTACT data. Record generation involves a two-step computational workflow: (i) the MSMS workflow, and (ii) the generation of MassBank records. In the first step, MS/MS data is extracted from the raw files, which are then recalibrated, denoised, and annotated with sub-formulas using the RMassBank procedures.^31^ The second step entails the annotation of peak lists using various web services and the settings file provided by the user. The SMILES in the input CSV is used to query information from the databases, *via* the InChIKey. The SMILES are converted into the InChIKey using CACTUS.^31^ The InChIKey is then used to retrieve synonyms, IUPAC names, and other identifiers from the Chemical Translation Service and PubChem.^31^ This is streamlined with the chemical compound information provided in the input compound lists and included in an output file and the corresponding MassBank records.

### Record validation with ReSOLUTION and MassBank validator

Once MassBank record files were generated, a quality check was carried out using the ReSOLUTION package. ReSOLUTION is an R package that contains functions to summarize the records, giving an overview of different fields within the text files. The getMBRecordInfo function of the ReSOLUTION package produces a CSV file that contains entries from all MassBank records within the specified directory that match the specified list of MassBank record fields. The summary file was manually verified to remove any spectra with a base peak intensity below 10^5^ and check for any other artefacts.

Following that, an additional validation step was performed to check if the records met the MassBank data criteria, as a part of pre-submission checks. The directory was set up for validating the records by downloading data from the MassBank-data repository on GitHub and the MassBank validation was performed using a bash script. Once all checks were passed, the spectra were submitted to MassBank *via* a GitHub pull request.

### MassBank–PubChem integration

The ReSOLUTION-based record validation was modified to extract data from the entire MassBank-data repository and produce summary files to enable the integration of MassBank.EU record previews in individual compound records in PubChem. After several rounds of display optimization with PubChem, the following fields were agreed upon (where available): Accession ID, Authors, Instrument, Instrument Type, MS Level, Ionization Mode, Ionization, Collision Energy, Fragmentation Mode, Column Name, Retention Time, Precursor *m*/*z*, Precursor Adduct, License, Publication, SPLASH (the SPectraL hASH^39^) and the Top-5 Peaks for display purposes, in addition to the Name, SMILES, InChI, InChIKey fields for structural information (to map to the correct PubChem record). Compound class information is also extracted but not currently used. The Top-5 peaks were chosen as the optimal display experience, as the Top-3 were determined to be too few, while the Top-10 created many overlaps in the spectral images and resulted in a lengthy data display. Trimming was also necessary to avoid issues with spectra that contained hundreds of peaks.

The entire MassBank summary file retrieved *via* ReSOLUTION forms the basis to create a substance file for deposition within PubChem. This takes the compound fields (SMILES, InChI and InChIKey), performs several clean-up steps (*e.g.*, removing “N/A” entries, SMILES with wildcards, and duplicate entries), and creates a file for deposition. During this process several issues were found with both MassBank.EU entries and the PubChem deposition checks. All errors in MassBank that could be addressed were corrected after discussion with contributors (see MassBank-data issues), while several updates were also made to the PubChem deposition process. Some MassBank entries had invalid SMILES that would not pass the improved deposition system, but could not be fixed due to conditions on the contributed MassBank record. These SMILES were added to a “bad list” and are removed during creation of the deposition file. The code for all these steps is available on the ECI GitLab repository^40^ (see “Data availability”).

Once the MS/MS and substance files were created, they were uploaded to Zenodo.^41^ An FTP service was set up to transfer the substance file automatically to PubChem when a new file is available. This data deposition can also be uploaded manually. The annotation is coordinated *via* a mapping file to the corresponding file on Zenodo, hosted on the ECI GitLab^40^ (see “Data availability”). Once a new MS/MS file is available it is parsed and updated at the next weekly cycle.

The spectra are rendered in PubChem on the fly with a simple CGI interface created as part of these efforts, but later expanded to display other contents in PubChem.^24^ The Top 5 peak display is designed to enable simple copy–paste into downstream applications. Major fields such as Accession ID and SPLASH are hyperlinked automatically to direct PubChem users back to the original MassBank data to retrieve the entire record.

## Results

### Prescreening of ENTACT mixtures

**Fig. 2:**
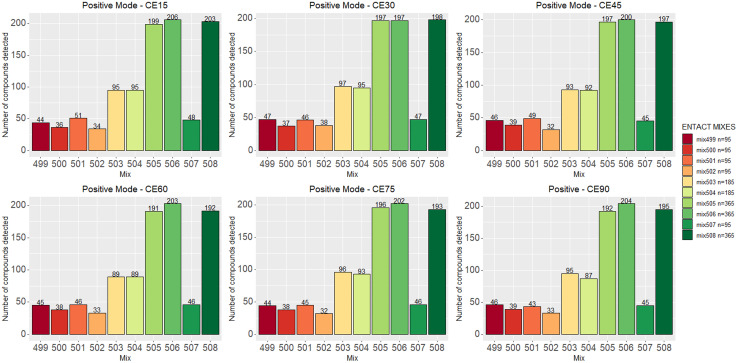
The number of compounds that pass the QC across multiple collision energies in positive mode. Negative data is given in Fig. S3.†

Fig. 2 shows the number of compounds that passed the quality control check at various collision energies for each mix in positive ESI mode. Fig. S3 (ESI†) shows the same for negative mode; the results are also summarized in Table S1.† The detection rate of each mode ranged between 45% and 55% per collision energy. As commonly observed in ESI, the number of compounds detected in positive mode was higher than in negative mode.

### MS/MS spectra record generation

The RMB-mix method for generating MS/MS spectral records was carried out on all the mixes. After recalibration and annotation, spectral records were generated for the compounds that passed the RMassBank workflow.

**Fig. 3:**
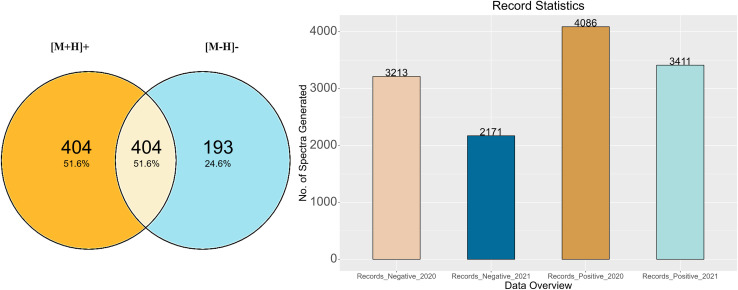
Summary of the record generation results. (A) The number of compounds generated in each mode and overlap. (B) Summary of MassBank records before (2020) and after (2021) isobar characterisation.

Fig. 3A shows the total number of compounds for which records were generated following the RMassBank workflow per mode (783 of the 1268 compounds in total), while Fig. 3B shows an overview of the corresponding number of spectral records that were generated (up to six records per compound and mode, due to the different collision energies). The records generated in the year 2020 included isobars (Records_Negative_2020 & Records_Positive_2020) in the compound list. Based on user feedback, this original data set contained ambiguous spectral records. As a result, the QA/QC procedures were tightened and isobaric substances were excluded. The workflow was rerun in 2021 to remove any ambiguities and to generate clean spectral records (Records_Positive_2021 and Records_Negative_2021). The procedure to remove ambiguities in the isobaric entries is explained in the next section with some examples.

### Characterisation of isobars

The results generated from the prescreening analysis were narrowed down to track the compounds with the same exact mass (isomers) or with a similar exact mass within the instrumental parameters (termed isobars for the purpose of this article). Isomers are compounds with the exact same mass but different structures. Isobars may not necessarily have the exact same mass but may be sufficiently close in exact mass that they are within the precursor isolation window of 1 Da and thus mixed spectra could be created if such isobars are present in the same mix with the same retention time.

**Fig. 4:**
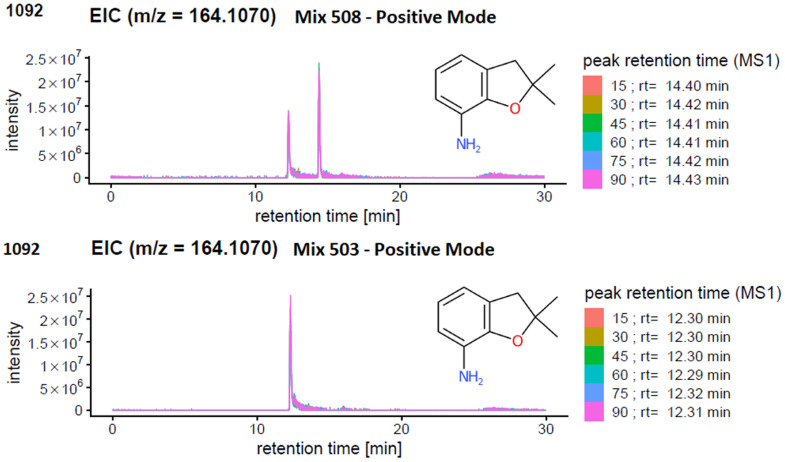
Extracted Ion Chromatogram (EIC) of *m*/*z* 161.1070 in (top) mix 508 and (bottom) mix 503. In mix 503 only one compound with this mass is present (level 1), but another isobaric compound is present in mix 508.

Since the ENTACT standards were mixtures, and the retention times (RTs) of the individual substances for the chromatographic system were not known, the presence of one or more isomers or isobars (see *e.g.*Fig. 4) in a given mix could give rise to:

(a) Distinct spectra for each isomer/isobar with separate RTs, without knowing which peak belongs to which isomer/isobar;

(b) Fewer spectra than isomers/isobars, without knowing which compounds could be detected;

(c) No spectra (trivial case – none of the compounds were detected).

Fig. 4 illustrates an example of an isobar in mix 503 and 508. One peak can be detected for the compound 2,2-dimethyl-2,3-dihydro-1-benzofuran-7-amine (*m*/*z* [M + H]^+^ = 164.1070 Da) in mix 503. However, screening for the same compound in (the more complex) mix 508 revealed one additional matching peak in the extracted ion chromatogram (EIC). Mix 508 contains both 2,2-dimethyl-2,3-dihydro-1-benzofuran-7-amine and *N*-(2,4-dimethylphenyl)acetamide, with the same formula C_10_H_13_NO. In this case, the presence of only one of the isobars in mix 503 helps determine that the retention time of 2,2-dimethyl-2,3-dihydro-1-benzofuran-7-amine must be 12.3 minutes (lower plot) while the retention time of *N*-(2,4-dimethylphenyl)acetamide must be the larger peak at 14.4 minutes. Thus, the spectra for 2,2-dimethyl-2,3-dihydro-1-benzofuran-7-amine could be extracted from mix 503 at 12.3 min (see PubChem CID 91697 and MassBank.EU MSBNK-LCSB-LU109201), while the spectra for *N*-(2,4-dimethylphenyl)acetamide could be extracted from mix 508 at 14.4 min (see CID 16303 and MSBNK-LCSB-LU121601).

**Fig. 5:**
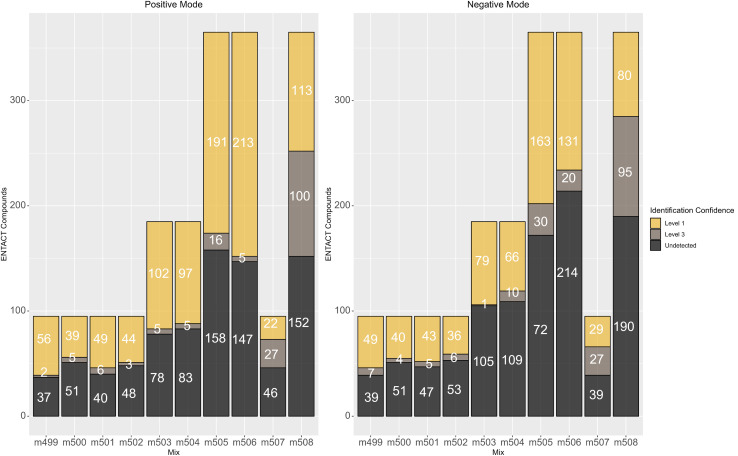
Number of non-detects (black), compounds that could be clearly identified (level 1, yellow) and isobars (level 3, mid-grey) in each mix.

In terms of reporting the results to the ENTACT organisers, all cases with more than one compound possible for more than one peak were reported as a level 3 confidence level (*e.g.*, Fig. 4, top case), whereas clear identifications (*e.g.*, Fig. 4, bottom case) were reported at level 1 confidence.^19^Fig. 5 summarises the identification levels of the compounds per mix and mode. Yellow indicates level 1 identifications (no isobars present), grey indicates level 3 (one or more isobars present), while black indicates the number of compounds not detected in that ionisation mode.

As observed above, there were generally fewer detections in negative mode, while the more complicated mixes 507 and 508 had proportionally more isobars than the simpler mixes. Mix 506 also had many level 3 identifications, especially in negative mode.

### Summary of the contribution to MassBank

The results of this study are summarized as follows: after prescreening using Shinyscreen, 750 compounds in positive mode and 587 compounds in negative mode met all the quality criteria, enabling the RMassBank workflow to be executed on these compounds prior to recalibration with the RMB mix method. The first set of 7299 spectral records (both positive and negative modes) was published on August 21, 2020. Upon characterization of the isobars in the mixes, ambiguous spectra were excluded from the database, leaving only clean spectra from 590 compounds in positive mode and 379 compounds in negative mode (including the overlap, see Fig. 3), which generated 3411 and 2171 spectral records respectively. The results of these analyses were updated in the MassBank database on 28 January 2021. In order to maintain the high quality of the MassBank database, this rigorous process ensured the quality and accuracy of all spectral records. The feedback from users also helped refine the quality control procedures applied to these complex ENTACT mixtures. The addition of 5582 spectral records was a valuable contribution to the public databases, given that the MassBank database contained only 519 ENTACT specific compounds (747 after), and the PubChem database contained only 479 of these ENTACT compounds in the LC-MS category before these efforts (780 after).

### PubChem–MassBank integration

**Fig. 6:**
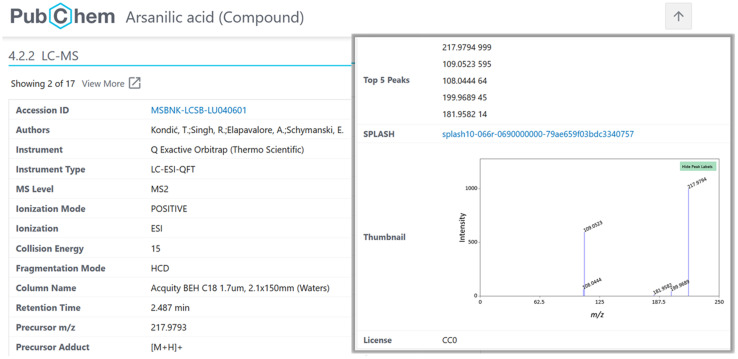
Example of a MassBank record displayed in PubChem (arsanilic acid, CID 7389), with the Accession ID and SPLASH hyperlinked to the original MassBank record and an interactive thumbnail.

**Fig. 7:**
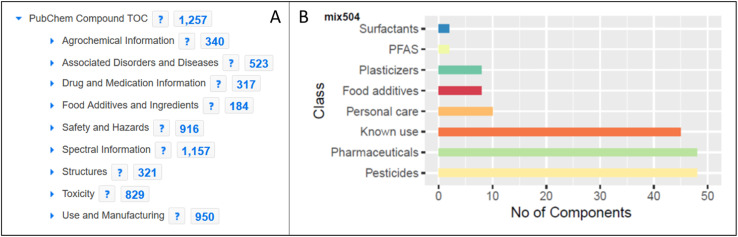
A) Exploring the entire ENTACT dataset on the PubChem table of contents (TOC) classification browser (https://pubchem.ncbi.nlm.nih.gov/classification/#hid=72). Note: some categories have been trimmed for space reasons. (B) Classification of mix 504 of the ENTACT dataset using PubChem annotation (sub)categories.

The inclusion of MassBank spectra in PubChem enabled the creation of spectral summary pages for each compound present in MassBank (see Fig. 6) and expanded both the collection of spectra in MassBank and the LC-MS category of data in PubChem (as discussed above). Since PubChem has millions of users a month, this helps provide an alternative way for researchers to find the spectral data in MassBank. It also enables the creation of powerful queries to explore available information about these compounds, as shown for mix 504 in Fig. 7.

## Discussion

### Data processing in R

The methodology and results specified in this study provide an insight into the systematic analysis of LC-MS data analysis and elaborate on the use of computational processing using R.^42^ R plays a significant role in cheminformatics and mass spectrometry *via* manipulating and analyzing chemical structures and data within the R environment. Many cheminformatics packages are available including rcdk, MSnbase, RMassBank and RChemMass, with several more packages available through CRAN,^43^ BioConductor and GitHub. Integrating the above packages into the R environment facilitates the development of new strategies in data handling, manipulation and implementation of a graphical user interface to serve as a platform for analyzing LC-MS data, preprocessing and visualization.^31^ Shinyscreen was developed in-house with flexibility and functionality, which excludes the need for programming experience in accessing the tools and can help overcome several of the inconveniences associated with package dependencies in R that can be exasperating for novice users. The key functional aspects of Shinyscreen most applicable to the work presented in this study include efficient data extraction, *i.e.*, exporting the data files to the interface and saving the configuration setting, providing initial quality control settings, visualizing the spectrum as a PDF file and saving the summary of results as a CSV file, all of which can be exported for further analysis. The efforts of manual prescreening using vendor software such as Xcalibur from Thermo can be minimized with the semi-automated Shinyscreen application. This is especially useful for complex datasets like ENTACT, because the preprocessing of data and visualizing the alignment of precursor and fragment ion retention times are simpler. The graphical user interface (GUI) for visualizing the MS1, MS/MS and peak alignment gives firsthand information on the compounds detected in the LC-MS instrument. Automatic quality control based on preset threshold values filtered out compounds failing the basic criteria accurately and there was no need for a subsequent manual check of eliminated results (only those remaining). Shinyscreen-assisted quality control was thus crucial for analyzing the quality of the dataset used by filtering out compounds failing the quality criteria before the RMassBank workflow. User feedback helped refine this process further, stimulating additional Shinyscreen developments.

The RMassBank workflow is independent of the Shinyscreen application. RMassBank is an R package that can be accessed through Bioconductor for the automatic recalibration and cleanup of spectral data due to noise or mass deviations. RMassBank can handle any number of files in one run relatively quickly, generating records and annotated compound information. Despite the semi-automated workflow successfully generating clean MassBank records, manual annotation and verification of the compound names retrieved *via* the web services is imperative before generating the records.

### Curation efforts

**Fig. 8:**
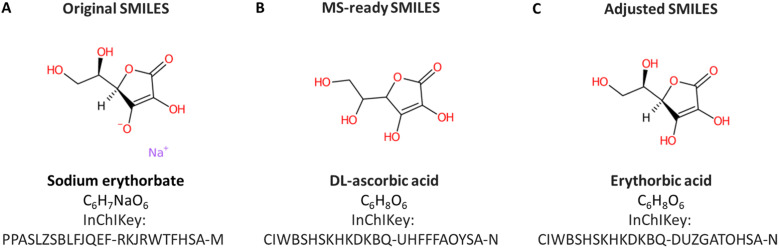
(A) Substance according to the original SMILES in ToxCast, sodium erythorbate. (B) The MS-ready version of the same compound, dl-ascorbic acid with salt and stereochemistry removed. (C) The compound according to the adjusted SMILES, restoring the correct stereochemistry – erythorbic acid.

Manual annotation of names for a large dataset like ENTACT is time-consuming and error-prone. Additionally, due to the way the data was provided by the US EPA, the SMILES used as input were MS-ready SMILES^29^ and thus the associated names were often the salt/mixtures and not the name associated with the final structure. A demonstrative example from the ToxCast set is shown in Fig. 8. The substance included in ToxCast was sodium erythorbate (Fig. 8A) with SMILES [Na^+^].OC[C@@H](O)[C@H]1OC(=O)C(O)=C1[O^−^], whereas the MS-ready SMILES provided by the US EPA was OCC(O)C1OC(=O)C(O)=C1O, matching the dl-ascorbic acid when performing the database retrieval (Fig. 8B). The correct match, with the same stereochemistry but without sodium and charges is erythorbic acid, with SMILES OC[C@@H](O)[C@H]1OC(=O)C(O)=C1O, shown in Fig. 8C. The fact that the MS-ready algorithm also removed stereochemistry hindered the recovery of information for many cases, since SMILES without stereochemistry were not always registered in databases for many cases (as they were not the complete/common representations). This made the retrieval of compound identifiers within the RMassBank workflow a challenge. To resolve this, an algorithm developed in-house helped retain the stereochemistry information from the original mixture SMILES provided with ENTACT. This issue was also reported to the US EPA for consideration in future MS-ready SMILES algorithm developments. A new infolist was generated to resolve the naming issue for these compounds. This was complemented by manual checking of the names to ensure correct information was present. In future efforts, it is likely that this workflow could be optimized further by adopting options like querying the PubChem database using InChIKeys and taking the PubChem title, IUPAC name, or top 3 synonyms, which could improve the handling of synonym issues in RMassBank.

Working with the ENTACT dataset was challenging due to the presence of isobars and isomers, which complicated the identification of chemicals. Due to unresolved peak annotations, several isobars were excluded from the results to ensure clean spectra in MassBank. For this reason, any isobars present were removed if the identity of the chemicals could not be determined (see *e.g.*Fig. 4). Ideally, to achieve accurate and clear analysis of data, individual standards should be used to distinguish each peak and effectively identify any isobars present in the sample, but in this case only the mixtures were available, not the individual ToxCast standards. These, however, have been made available to other ENTACT participants. Should this data also become publicly available, this would help further enhance the quality control procedures performed here and may also assist in identifying some of the spectra that have been removed due to uncertain identities at this stage. A good match between individual standard spectra and these spectra from mixtures could potentially assist in identifying isomers/isobars and help deduce their retention times on the chromatographic system applied here.

### Practical application of the ENTACT data

The 10 ENTACT combinations contain 1268 compounds, many of which are toxicologically relevant substances. The development of this in-house spectral library, which has also been uploaded to MassBank, was designed to aid in the identification of unknown compounds by improving the identification confidence *via* library match (level 2a) and even confirmation (level 1) with the retention time match if the same method was used. For external users of MassBank, this ENTACT dataset can provide level 2a, should acceptable match values be present. Since MassBank data is also integrated into other resources such as MassBank of North America^44^ and GNPS,^45^ as well as PubChem (as mentioned above), these spectra are now accessible to users worldwide. MassBank data is also integrated into MetFrag,^46^ so that this is also accessible to non-target workflows and other *in silico* methods.

The spectra uploaded as a part of this work have already supported other projects. This dataset served as an in-house reference library to identify pharmaceuticals^47^ and pesticides^48^ plus respective transformation products in Luxembourgish water samples with identification confidence level 1, supported by other individual reference standards. It was also used to complement spectral library data in exposomics work investigating Parkinson's disease.^49^ Several other studies in progress are also using this data. Hence, the 5582 spectral records generated in this study have already proven to be a valuable addition in setting up a mass spectral library in-house, and have added to the public databases as well.

In NTA research, the use of reference library spectra is frequently required for confident identification of unknowns. The number of possible identifications for each given study is limited due to the small quantity of available reference libraries.^50^ Creating a comprehensive chemical space is an enormous challenge, and to close the existing knowledge gap, it is imperative to share data across open access cheminformatics databases (PubChem,^24^ CompTox^25^) and repositories (NORMAN-SLE, Zenodo^51^). To ensure a continuous flow of information, a workflow has been established between PubChem and MassBank Europe, enabling deposition of spectral records into PubChem and integration of accompanying (relevant) annotation content with full traceability to original data sources.^20^ The code related to deposition and annotation can be found in the PubChem-MassBankEU GitLab repository^40^ and the files deposited on PubChem are available on Zenodo^41^ (see also “Data availability”). A key component of future efforts will be to continue to engage users and the community in developing tools and setting up databases that enable large compound knowledge bases for exposomics research, thus enabling researchers to interpret non-target HR-MS data in greater detail.^20^

## Conclusions

The exposome is a broad category and one of the major challenges in the non-targeted approach of exposome studies is the high complexity of data involved and the need for the development of consolidated MS/MS libraries for annotating the unknown compounds. This effort built a mass spectral library using the ENTACT dataset using different analytical and computational tools. The Shinyscreen and RMB-mix method packages ensured good quality MS/MS spectral record generation to set up the in-house spectral library. To date, ENTACT is one of the most extensive studies to use synthetic mixtures and multiple reference media samples to evaluate non-targeted approaches. Ideally, this will drastically improve the exposome research in exposure assessment, enhance the prediction and identification of risk factors and monitor policy results in reducing exposures.^1^ ENTACT data are toxicologically relevant and setting up a database of spectral records will help in the identification of unknown compounds. This is an important contribution for database search as there are few compounds available with public library spectra and not many ENTACT chemicals were in the database prior to these efforts. Together, these expansions will bring about an advancement in the non-targeted analysis. Although including ENTACT substances in the public record was a long-declared aim of the ENTACT organizers, the dataset committed as part of this study actually constitutes, to the best of our knowledge, the first public MS/MS dataset resulting from this trial. MassBank is currently preparing to accept further ENTACT contributions from other sources including the US EPA, which will enhance the use of this dataset by further contributing spectra measured on different instruments and under different conditions, further supporting robust identification efforts in exposomics.

## Abbreviations

APCI: Atmospheric pressure chemical ionization
CAS: Chemical abstract service
CE: Collision energy
CGI: Common gateway interface
CID: Compound identification
CSV: Comma-separated values
CTS: Chemical translation service
DOI: Digital object identifier
DSSTox: Distributed-structure-searchable toxicity
DTXCID: DSSTox structure identifier
DTXSID: DSSTox substance identifier
EC: Emerging contamiants
ECI: Environmental cheminformatics group (at LCSB)
EIC: Extracted ion chromatogram
ENTACT: EPA non-target analysis collaborative trial
ESI: Electrospray ionization
FTP: File transfer protocol
GC-MS: Gas coupled mass spectrometry
GNPS: Global natural products social molecular networking
GUI: Graphical user interface
HILIC: Hydrophilic interaction chromatography
HRMS: High resolution mass spectrometry
IUPAC: International union of pure and applied chemistry
InChI: International chemical identifier
InChIKey: IUPAC international chemical identifier key
LC-MS: Liquid chromatography mass spectrometry
LCSB: Luxembourg centre for systems biomedicine
MoNA: MassBank of North America
MS: Mass spectrometry
NIH: National institute of health
NTA: Non-targeted analysis
RPLC: Reverse phase liquid chromatography
RT: Retention time
(US) EPA: (United States) Environment Protection Agency

## Data availability

The MassBank records are available from the LCSB MassBank directory on GitHub or MassBank.EU. The packages and code are available on the ECI GitLab pages for Shinyscreen,^30^ RMB-mix^32^ and PubChem-MassBankEU.^40^ The files submitted to PubChem are archived on Zenodo.^41^ The ENTACT files have been uploaded to the GNPS database,^37^ including the raw files, the mzML files, compound lists for all of the ENTACT mixes, reporting template, settings file and the subsequent output files generated by Shinyscreen (MassIVE dataset number MSV000091754).

## Author contributions

A. E.: conceptualization, data curation, formal analysis, methodology, software, validation, visualization, writing – original draft preparation, writing – review & editing. T. K.: methodology, software, supervision, writing – review & editing. R. R. S.: methodology, writing – review & editing. B. A. S.: data curation, software, writing – review & editing. P. A. T.: data curation, software, writing – review & editing. J. Z.: data curation, methodology, software, visualization, writing – review & editing. E. E. B.: conceptualization, supervision, resources, writing – review & editing. E. L. S.: conceptualization, data curation, funding acquisition, software, supervision, resources, writing – original draft preparation, writing – review & editing.

## Conflicts of interest

The authors declare no competing interests.

## Supplementary Material

EM-025-D3EM00181D-s001
