# Supplementary material for: Adding open spectral data to MassBank and PubChem using open source tools to support non-targeted exposomics of mixtures
Source: Environ Sci Process Impacts. 2023 Jul 10;25(11):1788–801. doi: 10.1039/d3em00181d (PMC10648001; doi:10.1039/d3em00181d)
Supplement: EM-025-D3EM00181D-s001 [file EM-025-D3EM00181D-s001.pdf]

Electronic Supplementary Material (ESI) for Environmental Science: Processes & Impacts.
This journal is © The Royal Society of Chemistry 2023

Adding Open Spectral Data to MassBank and PubChem Using Open Source Tools to Support Non-Targeted Exposomics of Mixtures

Anjana Elapavalore[1]*, Todor Kondić[1], Randolph R. Singh[1,2], Benjamin A. Shoemaker[3], Paul A. Thiessen[3], Jian Zhang[3], Evan E. Bolton[3], Emma L. Schymanski[1]*

[1] Luxembourg Centre for Systems Biomedicine (LCSB), University of Luxembourg, 6 Avenue du Swing, 4367, Belvaux, Luxembourg. ORCIDs AE: 0000-0002-0295-6618; TK: 0000-0001-6662-4375; ELS: 0000-0001-6868-8145.

[2] IFREMER (Institut Français de Recherche pour l'Exploitation de la Mer), Laboratoire Biogéochimie des Contaminants Organiques, Rue de l'Ile d'Yeu, BP 21105, Nantes Cedex 3, 44311, France. ORCID RRS: 0000-0003-4500-3400.

[3] National Center for Biotechnology Information (NCBI), National Library of Medicine (NLM), National Institutes of Health (NIH), Bethesda, MD, 20894, USA. ORCIDs BAS: 0000-0002-1356-285X; PAT: 0000-0002-1992-2086; JZ: 0000-0002-6192-4632; EEB: 0000-0002-5959-6190.

* Correspondence: AE: anjana.elapavalore@uni.lu and ELS: emma.schymanski@uni.lu

Supplementary Information

This section contains three additional figures (Figures S1-S3) and a table (Table S1) in the following pages to support the article contents.

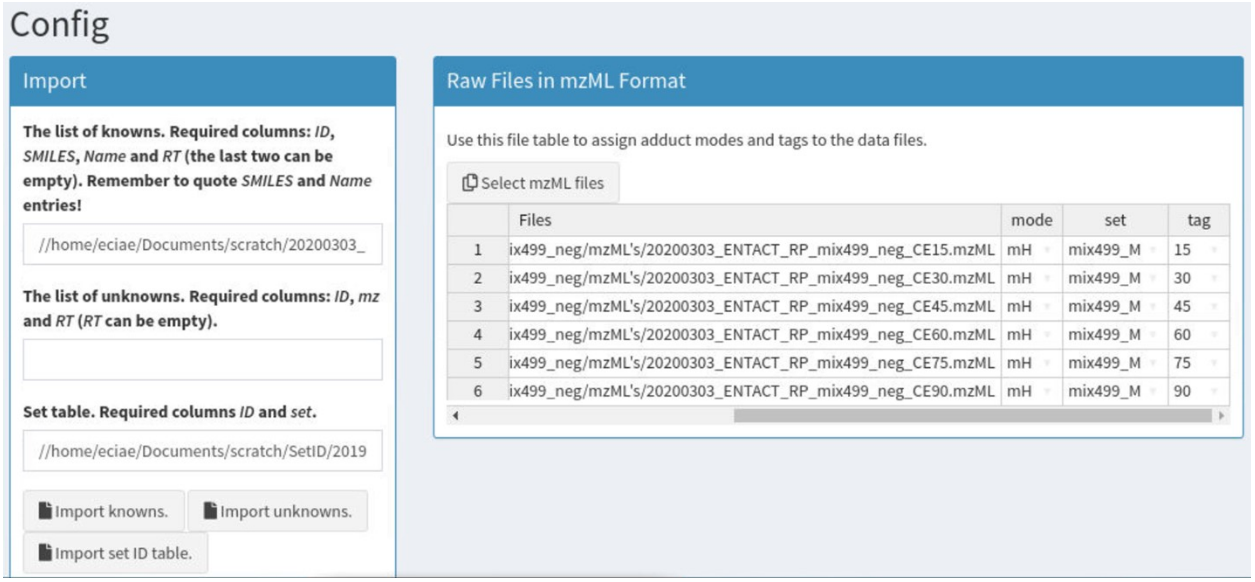

Figure S1: Shinyscreen Input Configuration.

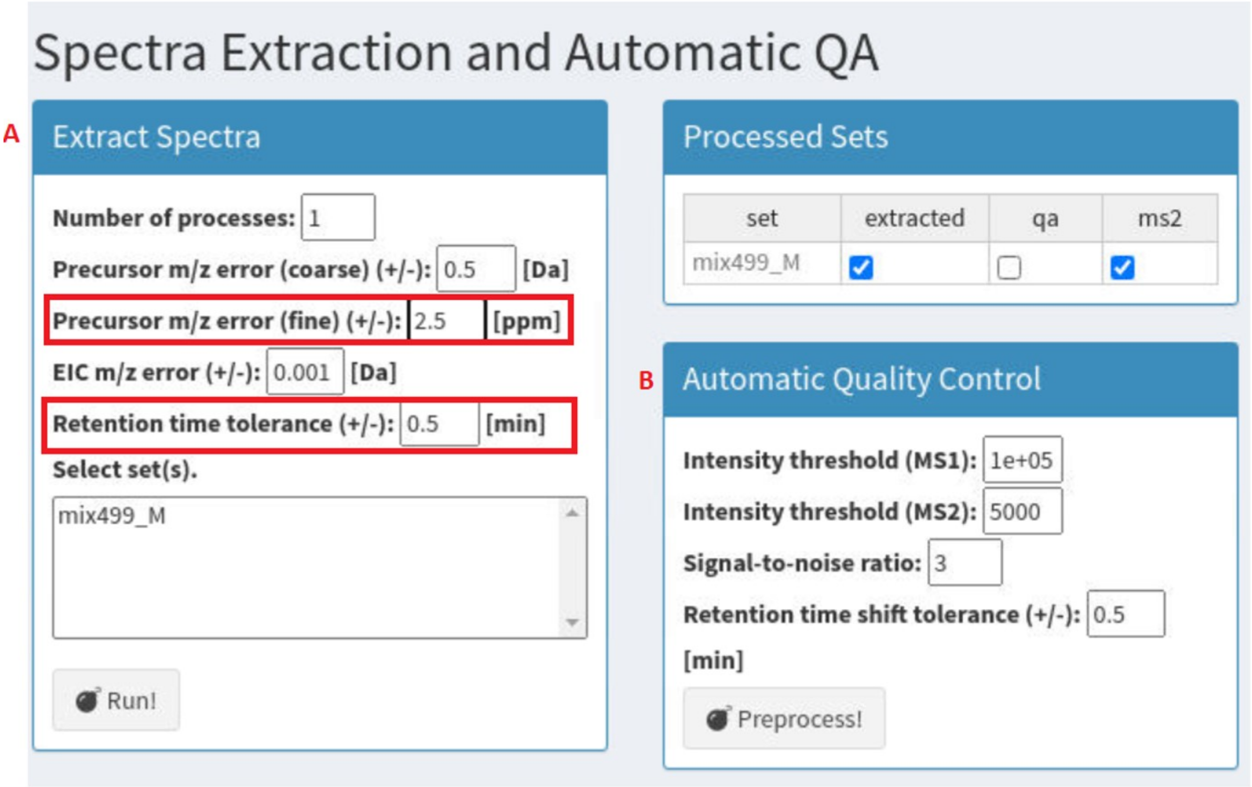

Figure S2: Spectral Extraction and Prescreening with Shinyscreen. A) Spectral Extraction Settings B) Automatic Quality Control Settings.

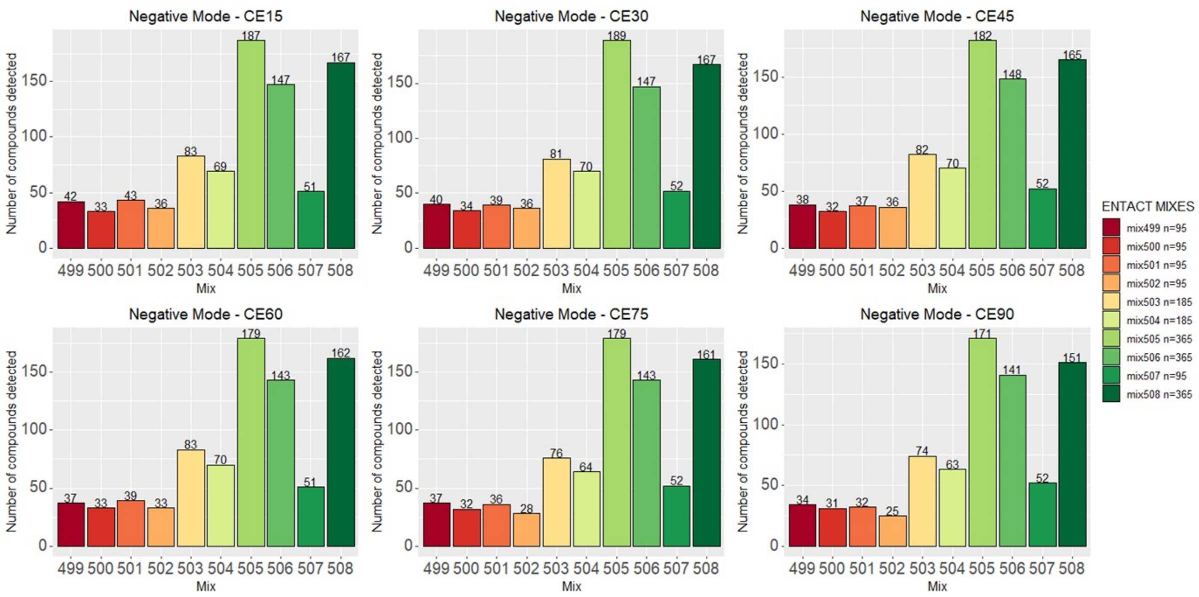

Figure S3: Prescreening results, negative mode.

Table S1: The number of compounds that passed quality control in Shinyscreen.

Mix	Total no. of compounds in each mix	No. of Compounds that passed the QC Positive mode						No. of Compounds that passed the QC Negative mode					
		CE15	CE30	CE45	CE60	CE75	CE90	CE15	CE30	CE45	CE60	CE75	CE90
499	95	44	47	46	45	44	46	42	40	38	37	37	34
500	95	36	37	39	38	38	39	33	34	32	33	32	31
501	95	51	46	49	46	45	43	43	39	37	39	36	32
502	95	34	38	32	33	32	33	36	36	36	33	28	25
503	185	95	97	93	89	96	95	83	81	82	83	76	74
504	185	95	95	92	89	93	87	69	70	70	70	64	63
505	365	199	197	197	191	196	192	187	189	182	179	179	171
506	365	206	197	200	203	202	204	147	147	148	143	143	141
507	95	48	47	45	46	46	45	51	52	52	51	52	52
508	365	203	198	197	192	193	195	167	167	165	162	161	151
